# Nonlinear Bivariate Associations and Mononuclear Cell‐Type‐Specific Expression Level Differences in the STING Signalling Pathway

**DOI:** 10.1111/jcmm.71093

**Published:** 2026-03-16

**Authors:** David Kaplan, Eric L. Christian

**Affiliations:** ^1^ CellPrint Biotechnology LLC Cleveland Ohio USA; ^2^ Case Western Reserve University Cleveland Ohio USA

**Keywords:** atherosclerotic coronary artery disease, linear bivariate correlation, nonlinear bivariate correlation, peripheral blood mononuclear cells, STING pathway

## Abstract

Understanding intracellular signalling pathways is crucial since they regulate essential functional activities. Bivariate relationships have been useful in delineating these pathways in clinical samples. In our previous studies, we have found many linear associations between pathway components, and we have interpreted these correlations as rheostatic regulators. Increases in an upstream component are correlated with a commensurate downstream increase. Here, we report a quantitative analysis of molecules in the STING pathway by assessing the variance in human peripheral blood mononuclear cell‐type‐specific molecular expression from patients with atherosclerotic coronary artery disease. The induction of the type I interferon track by this pathway is dependent on the expression levels of STING in T cells and monocytes and the expression levels of phospho‐STING in B cells. This relationship in T cells and monocytes demonstrates definitive linearity, indicating that it is regulated as a rheostat. In B cells the relationships are logarithmic, indicating an on‐off mechanism of regulation. The STING pathway‐dependent stimulation of the NFκB track is also controlled by on‐off mechanisms that are modelled by nonlinear bivariate relationships. These on‐off switches occur at the bifurcation of the two branches involving phospho‐STING, phospho‐TBK1 and phospho‐RelA. Whereas linear bivariate associations are readily captured by an evaluation of the correlation matrix, significant nonlinear relationships are not. Nonlinear correlations modelled logarithmically or exponentially are easily discerned by the assessment of a natural log‐transformed versus non‐transformed correlation matrix.

## 1 Introduction

The presence of DNA in the cytoplasm is aberrant and consequently serves as an indicator of a pathological situation such as infection, cancer or cellular damage. The STING (stimulator of interferon genes) pathway involves the detection of and response to cytoplasmic DNA. The outlines of the STING pathway have been determined through elegant experimental investigations with various technologies, including knock‐out mice, tumour cell lines, RNA interference and pharmacologic treatment [1, 2].

Cyclic guanosine monophosphate‐adenosine monophosphate synthase (cGAS) detects DNA in the cytoplasm and acts as an innate receptor initiating an immune/inflammatory response [1]. DNA bound to cGAS induces a conformational change that stimulates the production of cyclic GMP‐AMP, a high‐affinity ligand for STING. This binding event causes STING to activate TANK‐binding kinase 1 (TBK1) by oligomerisation leading to TBK1 autophosphorylation, and subsequently the STING‐TBK1 complex is responsible for the phosphorylation of interferon regulatory factor 3 (IRF3), which induces the production of type I interferon [1]. STING‐TBK1 also activates NFκB, which leads to the production of a distinct set of inflammatory mediators: TNFα, IL1β and IL6 [3, 4, 5].

STING is directly activated by phosphorylation on serine 366 in short‐term culture [6, 7]. Nevertheless, phosphorylation at this same residue has also been shown to be inhibitory by autophagic degradation within twelve hours [8]. Consequently, the STING pathway involves a negative feedback mechanism involving STING phosphorylation that serves to inhibit the activation of the pathway.

The induction of the inflammatory response by STING is a medically important pathway involved in various human disorders including myocardial infarction, cancer, rheumatoid arthritis, systemic lupus erythematosus and age‐related macular degeneration [1, 2, 9, 10]. We have developed a high‐resolution flow cytometric technology with restricted dimensionality and signal amplification [11, 12, 13, 14, 15, 16, 17, 18, 19, 20, 21, 22] and have shown that our technology recapitulates the tenets of the STING pathway in mononuclear cells stimulated in culture with a STING agonist [22].

In this study, we collected peripheral blood from 120 patients with risk factors for atherosclerosis exhibiting a wide spectrum of clinical manifestations. Consequently, we relied on the variance in samples from patients with varying degrees of atherosclerotic disease and inflammatory pathology to interrogate the relationships among the various components of the STING pathway. The rationale for the study was to assess the pathway in mononuclear cells from clinical samples without experimental pathway stimulation.

## 2 Methods

### 2.1 Cell Donors

All blood donations were obtained after informed consent. Sample collection and cellular analysis were approved by the Institutional Review Board of Case Western Reserve University School of Medicine. The study was conducted in accordance with the International Conference on Harmonisation Guidelines for Good Clinical Practice and the principles of the Declaration of Helsinki. For the initial set of samples, donors were patients being evaluated or managed for coronary artery disease at University Hospitals Cleveland or Louis Stokes Cleveland Veteran Affairs Medical Center. The samples were provided by Dr. David Zidar of Case Western Reserve University. For the initial study 120 de‐identified viably frozen blood mononuclear cell samples were obtained. Because the cells in some of the samples demonstrated low viability and because the cells in some of the samples were inadequate for the complete analysis, the number of samples included in the study varied as follows: for CD4^+^ T cells *n* = 115; for CD8^+^ T cells *n* = 99; for CD19^+^ B cells *n* = 108; and for monocytes *n* = 115. Elimination of samples from analysis was determined prior to any analysis being commenced. For the confirmatory cohort, 55 viably frozen samples were obtained. Approximately half of them were derived from the samples previously analysed in the initial study, and the other half included samples from patients with symptomatic coronary artery disease. Since the patients participating in the initial study did not present with symptomatic disease, the patients in the confirmatory cohort displayed a greater range of disease activity than the original sample.

### 2.2 Cells

Mononuclear cells were isolated from blood samples by discontinuous gradient centrifugation over ficoll/hypaque. The cells were viably frozen in 10% dimethyl sulfoxide and stored under liquid nitrogen until use.

### 2.3 Reagents

Primary antibodies with specificities for STING (Thermo, cat# MA532768), phospho‐STING ser 366 (Cell Signalling Technology: CST, cat# 40818), IRF3 (Abcam, cat# 68481), NLRP3 (Thermo, cat# MA532255), BDNF (Abcam, cat# ab108319), phospho‐Akt thr308 (CST, cat# 2965), phospho‐TBK1 ser172 (CST, cat# 5483), phospho‐RelA ser536 (CST, cat# 3033), phospho‐ULK1 ser757 (CST, cat# 14202), Traf6 (Abcam, cat# ab33915) and MyD88 (Abcam, cat# ab133739) were obtained from commercial sources. For the confirmatory study, primary antibodies with specificities for phospho‐GSK3β ser9 (CST, cat# 5558) and phospho‐IKKε ser172 (Bioss, cat# bs8583R) were also purchased.

### 2.4 Restricted Dimensional Cytometric Analysis (rdC) With Signal Amplification

Cells were quickly thawed prior to analysis, stained with lineage markers (CD4 for CD4^+^ T cells and monocytes, CD8 for CD8^+^ T cells and CD19 for B cells) in separate tubes and fixed in paraformaldehyde, followed by permeabilisation with saponin. Co‐stains were obtained from BioLegend. Amplification staining and restricted dimensionality were accomplished as previously described [11, 12, 13, 14, 15, 16, 17, 18, 19, 20, 21, 22]. Briefly, the cells were incubated with primary antibodies at room temperature and washed thoroughly. The cells were incubated with either anti‐rabbit or anti‐fluorescein antibodies conjugated with horseradish peroxidase at room temperature. After thorough washing, the cells were incubated with hydrogen peroxide and fluoresceinated tyramide at room temperature. After thorough washing, the cells were analysed on a BD Accuri flow cytometer, and the results analysed with FlowJo software. The average number of events per peak was 6246. The analysis was performed with the technologists and analysts blinded to the diagnosis associated with the de‐identified samples and to all other clinical data.

### 2.5 Statistical Analysis

ANOVA and Pearson correlational coefficients were calculated with SPSS and Excel software. Nonlinear bivariate correlations (exponential, logarithmic, power) were modelled by Excel software.

## 3 Results

### 3.1 STING Pathway Molecules Are Differentially Expressed in Mononuclear Cells

Mononuclear cells from 120 patients with risk factors for atherosclerosis were isolated and tested for expression of eleven analytes related to the STING pathway: STING, phospho‐STING (ser366), phospho‐TBK1 (ser172), phospho‐RelA (ser536), IRF3, NLRP3, BDNF, phospho‐Akt (thr308), phospho‐ULK1 (ser757), Traf6 and MyD88. Type I interferon, which is rapidly secreted and thereby not a good candidate for our study, stimulates BDNF production by mononuclear cells [23, 24, 25, 26]; consequently, BDNF was included as a surrogate indicator of type I interferon production. Since BDNF activates the Akt pathway [27, 28], we also assessed phospho‐Akt expression in our analysis.

**FIGURE 1:**
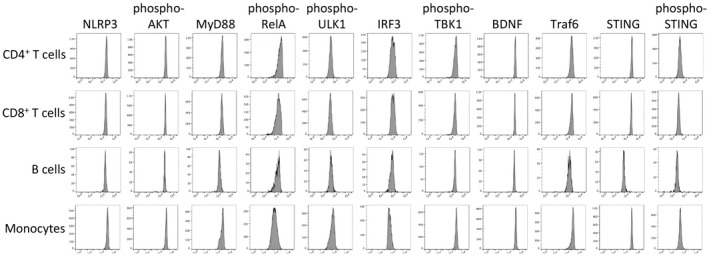
Representative histograms for each analyte and each cell type demonstrates definitive expression levels.

Representative examples of the histograms for expression stratified by cell type are shown in Figure 1. The peaks of expression are unimodal with limited variance, indicating that single values reasonably represent expression levels.

**FIGURE 2:**
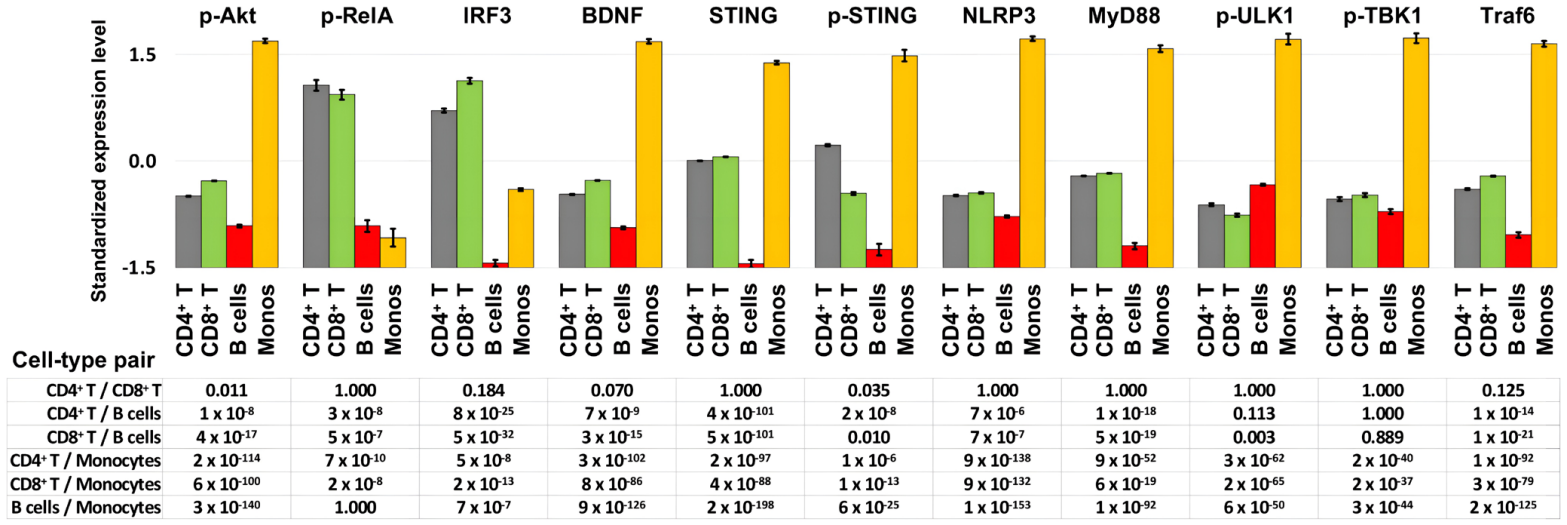
Cell‐type‐specific expression levels of STING pathway components. Expression was assessed by signal amplification and flow cytometry, and median expression levels, as indicated by fluorescence intensity, were converted to Z scores per analyte to facilitate comparisons. The prefix p in analyte names indicates the phosphorylated substrate. Error bars represent the standard error of the mean. Under each plot, the ANOVA analyses are shown for each pair of cell types. The *p*‐values shown have been adjusted by the Bonferroni correction for multiple comparisons.

The cell‐type‐specific expression of the eleven analytes (Figure 2) shows significant differences among the major mononuclear cell types. For instance, the antigen‐presenting cells (monocytes and B cells) express less phospho‐RelA and IRF3 than the T cells. Strikingly, B cells express relatively low levels of STING and IRF3 compared to the other cell types. CD4^+^ and CD8^+^ T cells demonstrated similar expression levels for most of the analytes. These results indicate that the STING pathway is uniquely configured among the major mononuclear cell types.

### 3.2 Linear and Nonlinear Associations Among STING Pathway Molecules

**FIGURE 3:**
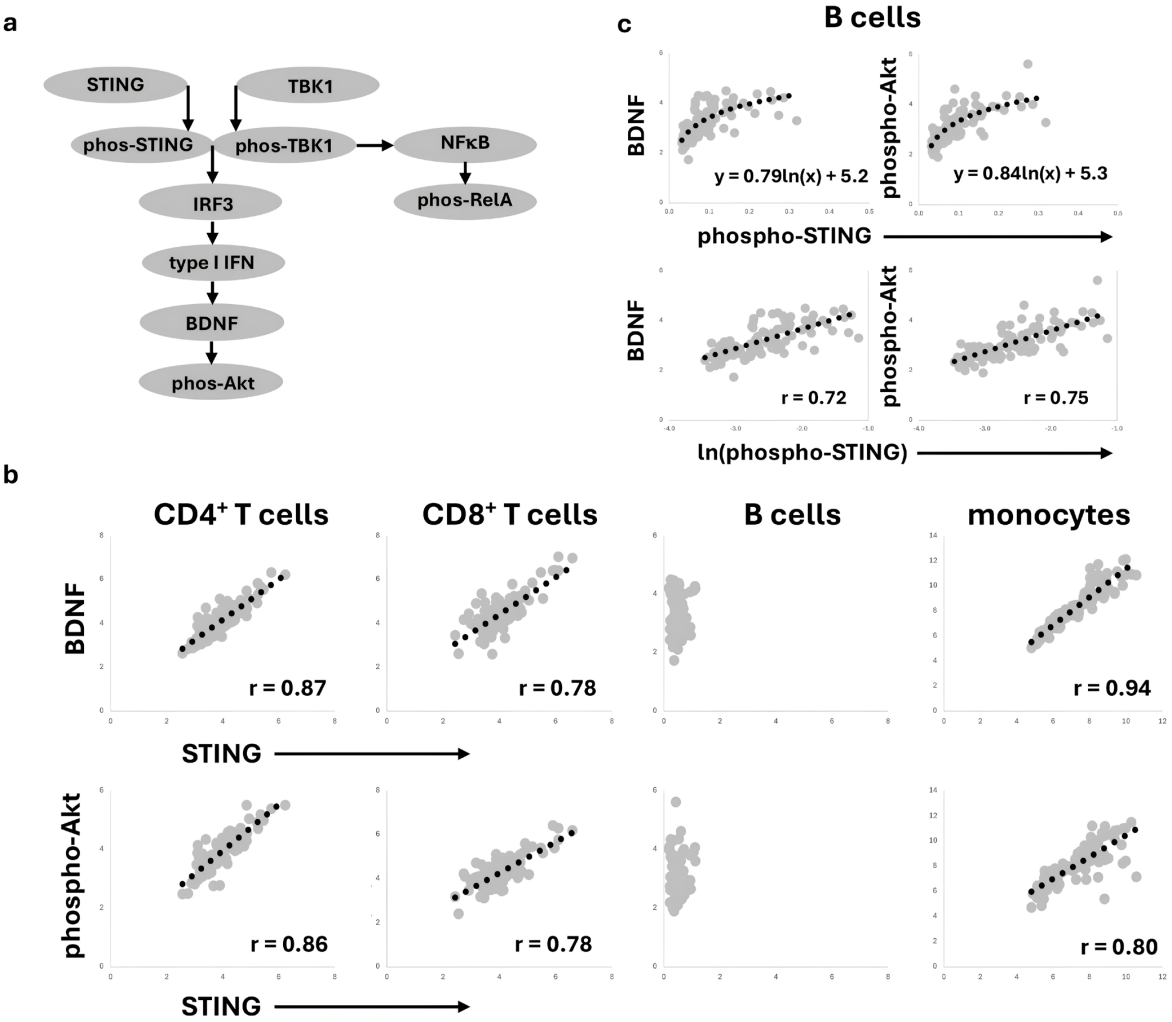
Cell‐type‐specific regulation of the type I interferon branch of the STING pathway. Bivariate expression levels are shown. (a) STING pathway model showing the type I interferon and the NFκB branches. (b) Bivariate relationships of STING with BDNF and phospho‐Akt are plotted for the various mononuclear cell types. Linear regressions with corresponding r values are shown. (c) Bivariate relationships of phospho‐STING with BDNF and phospho‐Akt are plotted for B lymphocytes. Logarithmic regressions are shown with the corresponding equation in each upper panel. The lower panels plot the BDNF and phospho‐Akt with the natural log transformation of phospho‐STING with the linear regression and correlation coefficients shown in each panel.

The correlation matrix of our data revealed sets of molecules that exhibit definitive linear relationships (Figure 3). Correlations do not provide definitive causal information, but causation in the STING pathway has been previously established in experimental systems [1, 2].

Most importantly, with BDNF and phospho‐Akt as indicators of the activity of the type I interferon track of the STING pathway (Figure 3a), we found that the expression levels of these two molecules were significantly correlated with STING for both T cell subsets and monocytes, and the association was linear as indicated by the regression and the Pearson correlation coefficients (Figure 3b). The level of STING expression is exceptionally low in B cells (Figure 2) and does not correlate with the other components of the pathway. However, phospho‐STING in B cells does significantly correlate with BDNF and phospho‐Akt (Figure 3c), and the correlation fits a logarithmic pattern as demonstrated with the linearity of the plot of both BDNF and phospho‐Akt with the natural logarithm of phospho‐STING (ln(phospho‐STING)).

**FIGURE 4:**
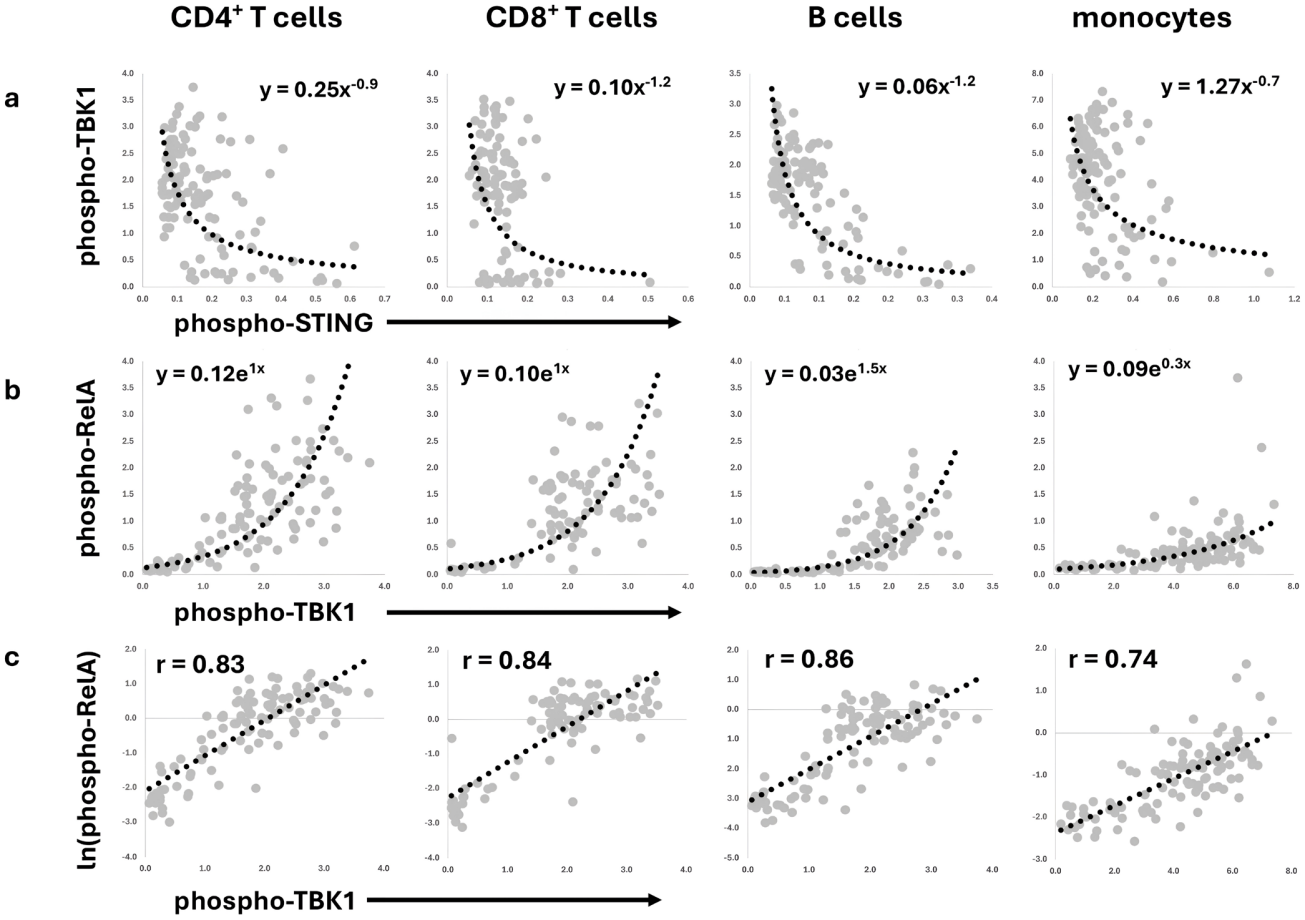
Cell‐type‐specific bivariate relationships between phospho‐TBK1, phospho‐STING and phospho‐RelA. (a) The bivariate expressions of phospho‐TBK1 and phospho‐STING are shown with inverse power regression and the corresponding regression equation. (b) The bivariate expressions of phospho‐TBK1 and phospho‐RelA are shown with exponential regression and the corresponding regression equation. (c) The bivariate expression of the natural logarithmic transformation of phospho‐Rela and phospho‐TBK1 is shown with linear regression and the corresponding r values.

In the correlation matrix, we also noticed significant negative correlation coefficients between phospho‐STING and phospho‐TBK1 (Table S1). The complex of phospho‐STING and phospho‐TBK1 occurs at the bifurcation of the type I interferon and the NFκB tracks of the STING pathway (Figure 3a). The bivariate plot of these two elements was not optimally modelled with an inverse linear relationship, but instead showed a striking inverse power association (Figure 4a). The possible association of phospho‐TBK1 and phospho‐STING was especially interesting to consider in light of the known interaction of these molecules.

**FIGURE 5:**
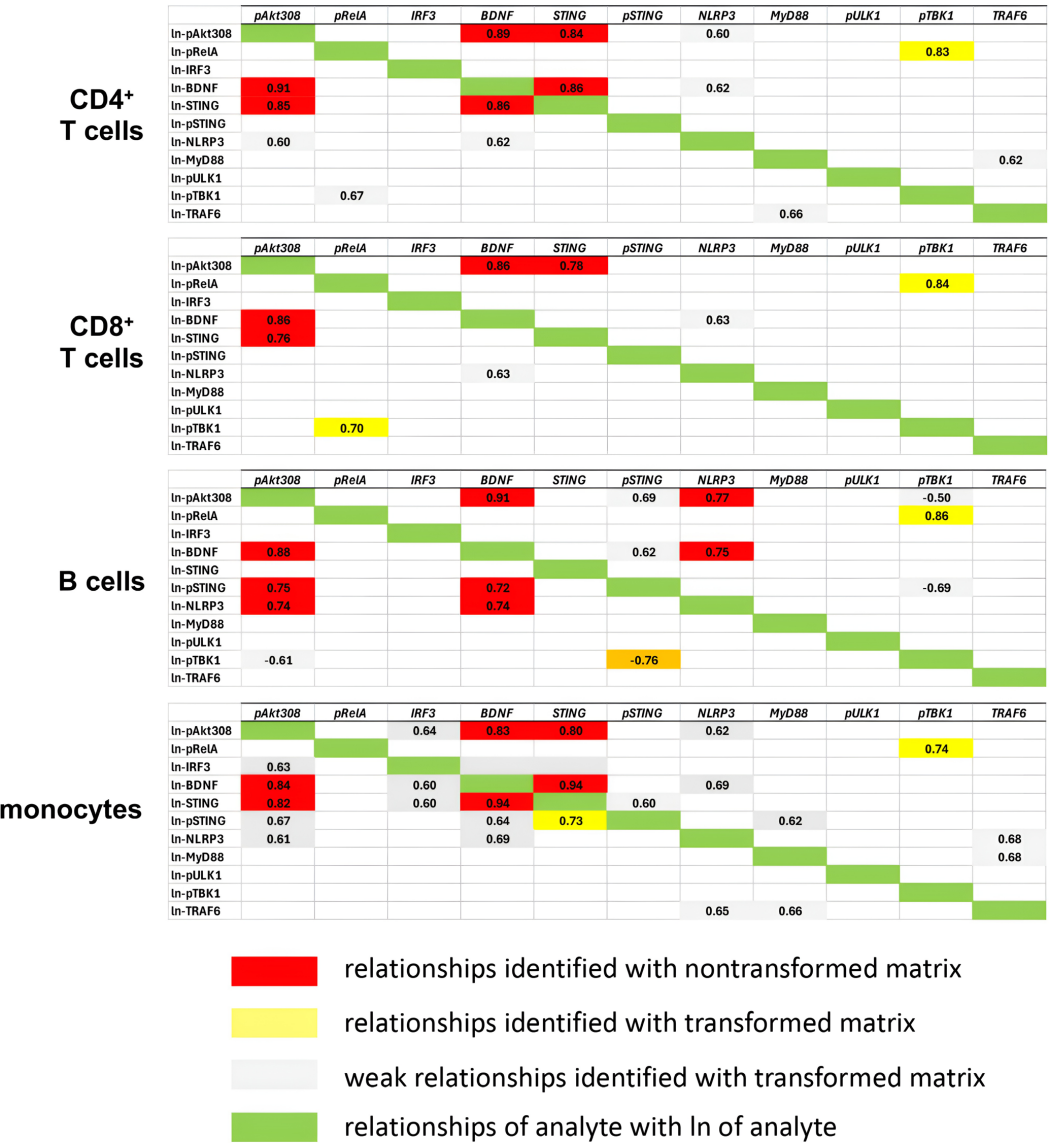
Non‐transformed versus ln transformed analyte correlation matrices for each mononuclear cell type are presented as a heat map. The bivariate relationships with *r* values > 0.6 and < −0.6 are shaded. Weak relationships are defined as correlations with 0.6 < r < 0.7 or − 0.6 > r > −0.7. Note that the correlation coefficient for ln(x) and y is not necessarily identical to the coefficient for x and ln(y). Also, the correlation coefficients for x versus ln(x) (signified by green fill in the figure) were > 0.9 with a single exception.

Having found a nonlinear, logarithmic bivariate relationship between phospho‐STING and both BDNF and phospho‐Akt in B cells, we looked for additional nonlinear associations with a non‐transformed versus natural log‐transformed correlation matrix for each cell type (Figure 5). We noted a strong relationship between phospho‐TBK1 and ln(phospho‐RelA) in all mononuclear cell types in comparison to their potential association in the non‐transformed correlation matrix (Table 1). The relationship between phosphorylated TBK1 and phosphorylated RelA fit an exponential model (Figure 4b), validated by the linearity of the plot of phospho‐TBK1 and the natural logarithm of phospho‐RelA (ln(phospho‐RelA); Figure 4c).

**TABLE 1:** Comparison of the non‐transformed correlation matrix to the non‐transformed versus natural log‐transformed correlation matrix for modelling the phospho‐TBK1:phospho‐RelA relationship.

Cell type	n	phospho‐TBK1 phospho‐RelA	phospho‐TBK1 ln(phospho‐RelA)	*p* ^a^(r‐to‐z)
CD4^+^ T cells	115	r = 0.72; *p* = 10^−19^	r = 0.83; *p* = 10^−30^	0.0357
CD8^+^ T cells	99	r = 0.72; p = 10^−17^	r = 0.84; p = 10^−27^	0.0300
B cells	108	r = 0.68; p = 10^−15^	r = 0.86; p = 10^−33^	0.0008
monocytes	115	r = 0.44; p = 10^−6^	r = 0.74; p = 10^−21^	0.0003

^a^ The *p* values were calculated for the comparison of the phospho‐TBK1:phospho‐RelA association versus phospho‐TBK1:ln(phospho‐RelA) after Fisher's r‐to‐z transformation. The results shown have been stratified by mononuclear cell type.

**FIGURE 6:**
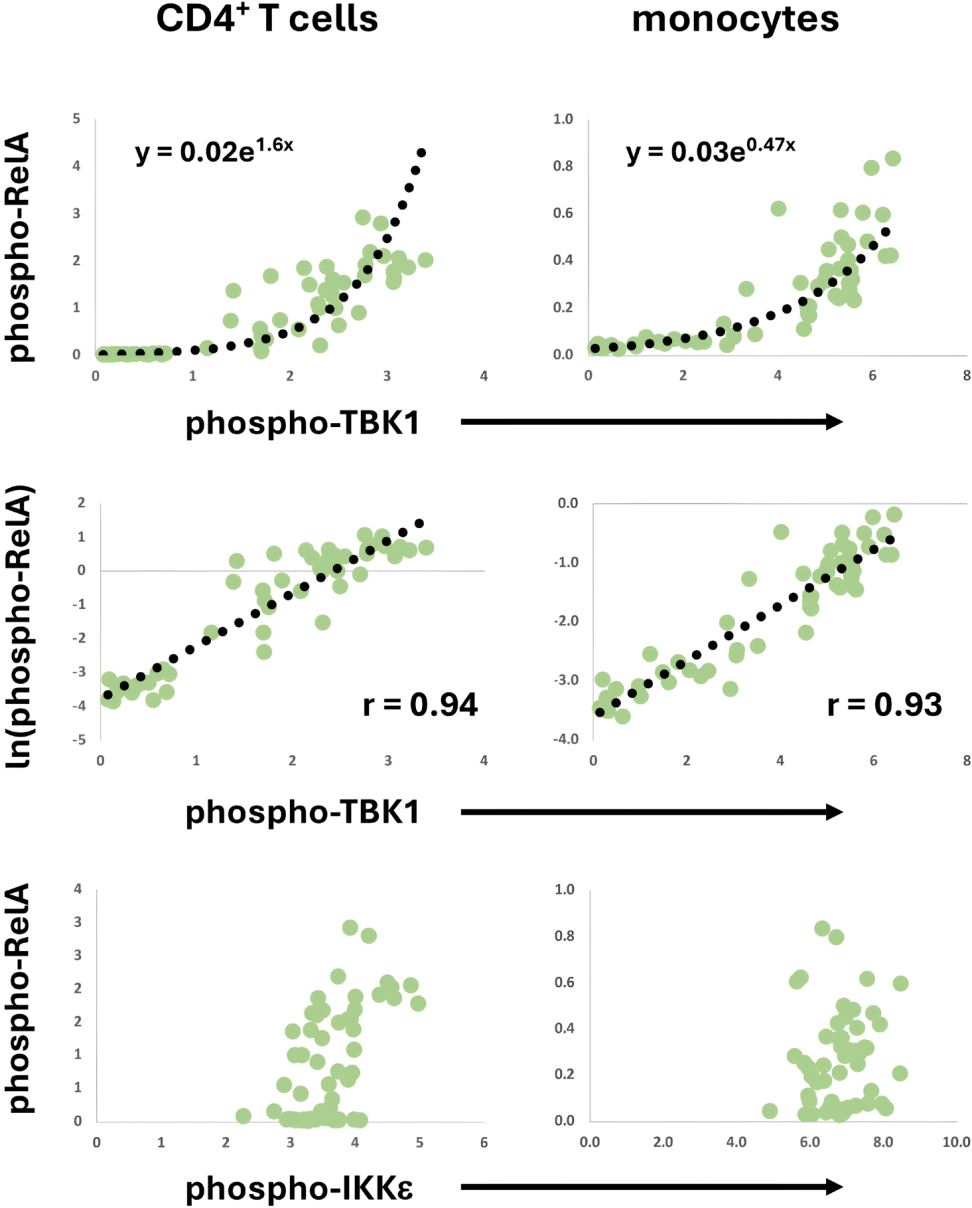
Cell‐type‐specific bivariate relationships between phospho‐TBK1 and phospho‐RelA. Additional aliquots of samples from 55 patients were assessed for the expression of phospho‐TBK1, phospho‐RelA and phospho‐IKKε in CD4^+^ T cells and monocytes. The bivariate expressions of phospho‐TBK1/phospho‐RelA and phospho‐TBK1/ln(phospho‐RelA) are shown with exponential trendlines. Exponential equations are shown in the top panels, and the correlation coefficients for the natural log‐transformed phospho‐RelA and phospho‐TBK1 relationships are shown in the middle panels.

The exponential relationship between phospho‐TBK1 and phospho‐RelA was confirmed in an analysis of a related cohort (*n* = 55 with 27 samples overlapping with the previous cohort and 28 samples unique) in CD4^+^ T cells and monocytes (Figure 6). Additionally, the specificity of this pattern was assessed by including phospho‐IKKε in the analysis since this molecule is an IκB kinase family member closely related to TBK1 and also known to phosphorylate RelA [29]. The results show the specificity of the association since phospho‐TBK1 but not phospho‐IKKε show a direct logarithmic correlation with phospho‐RelA.

In the second analysis, we included an assessment of phospho‐GSK3β expression because of the known association between GSK3β and TBK1 [30, 31]. TBK1 phosphorylates GSK3β [30], and GSK3β induces TBK1 autophosphorylation in a way that is not dependent on the kinase activity of GSK3β [31]. A positive linear association of phospho‐TBK1 and phospho‐GSK3β was observed in the analysis of the second cohort in both CD4^+^ T cells and monocytes, whereas the relationship between phospho‐IKKε and phospho‐GSK3β was considerably attenuated (Figure S1).

### 3.3 Comparing STING Pathway Molecular Associations In vitro and Ex vivo

**FIGURE 7:**
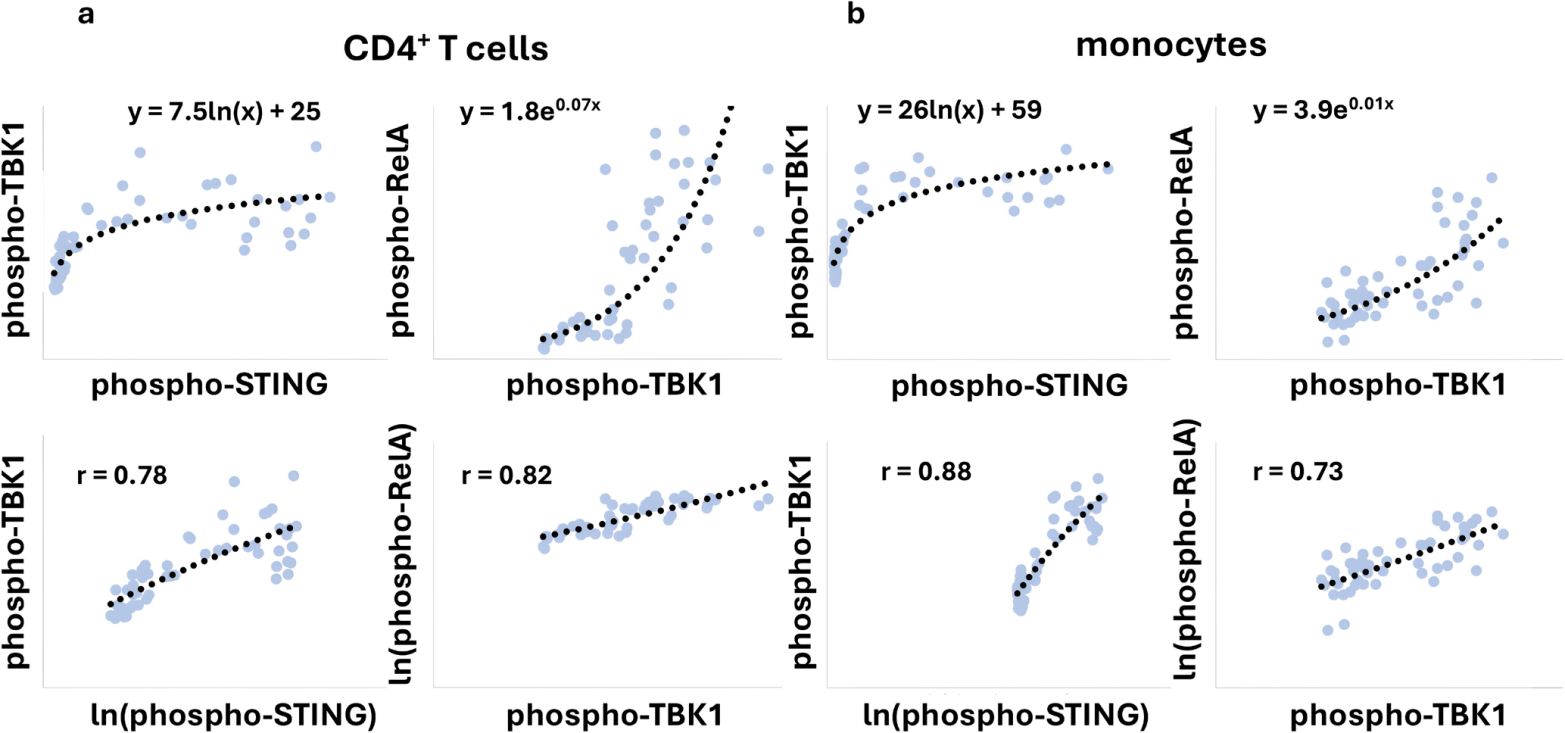
STING agonist affects pathway components. Peripheral blood mononuclear cells from 6 healthy volunteers were treated with varying concentrations of the STING agonist diABZI (0 and 5 5‐fold dilutions from 1 μM) for one hour in culture medium at 37°C in an atmosphere of 5% CO_2_. (a) Phospho‐STING, phospho‐TBK1 and phospho‐RelA expression levels were analysed in (a) CD4+ T cells and (b) monocytes. Equations are shown for logarithmic and exponential models (upper panels). Log normal (Ln) transformations were performed for phospho‐STING and phospho‐RelA (lower panels), and the correlation coefficients (*r*) are shown.

Because we were surprised by the inverse power relationship between phospho‐STING and phospho‐TBK1, we probed this bivariate association in an in vitro experimental model with cultured mononuclear cells from six healthy donors treated with varying concentrations of the STING agonist di‐amidobenzimidazole (diABZI; [32]) for one hour and tested for the expression of the phosphorylated molecules (STING, TBK1 and RelA) associated with the various cell types (Figure 7).

For this analysis, the varying concentrations of diABZI induced variance in the expression levels of the three phosphoantigens. A logarithmic relationship was seen in both cell types between phospho‐TBK1 and phospho‐STING (Figure 4, left panels in a and b). This result was distinct from the results in the ex vivo study (Figure 3a) which may be explained by the duration of the stimulus. The ex vivo study is an assessment of the cumulative in vivo regulatory mechanisms which are not necessarily reflected in the short‐term in vitro study.

With the STING agonist in vitro, phospho‐TBK1 and phospho‐RelA exhibited an exponential relationship (Figure 4, right panels in a and b), which is similar to the relationship seen in the ex vivo study (Figures 3b and 3c).

## 4 Discussion

We have previously used high‐resolution flow cytometry with restricted dimensionality to delineate signalling pathways in mononuclear cells from patients [14, 15, 16, 17, 18, 19, 20, 22]. This approach relies on exceptional sensitivity to detect low‐abundance analytes and stringent quality control to assure precise determination of expression levels. Pathways have been discerned by mapping bivariate associations with our platform in studies of patients with bipolar disorder [16, 17, 18, 19], AL amyloidosis [22] and chronic lymphocytic leukaemia [14] as well as patients treated with G‐CSF to mobilise hematopoietic cells from the bone marrow [20] and mononuclear cells treated in culture with a Bcl2/BclxL/Bclw inhibitor [22].

We have previously used our technology platform to assess the STING pathway after short‐term in vitro stimulation with an appropriate agonist [22]. In this study, we evaluated the components of the STING pathway by interrogating human mononuclear cells ex vivo and relying on the variance of STING pathway molecular expression in patients being evaluated for coronary artery atherosclerosis.

With our approach, we have shown that the pathway is configured uniquely in different mononuclear cell types. B lymphocytes express significantly less STING and IRF3 than T cells or monocytes. The absence of STING expression in human B cells was previously observed by western blotting and RT‐PCR after transfection of the cells with double‐stranded DNA [33]. In our study, B cells were neither transfected nor cultured, and although the level of STING was low, it was not totally absent.

STING expression levels in T cells and monocytes, but not in B cells, correlated with the expression of downstream pathway components of the type I interferon track, BDNF and phospho‐Akt. In B cells, phospho‐STING, not STING, is correlated with these downstream pathway components. The reason for the unique configuration of the STING pathway in B cells may be related to the low level of STING expression in these cells.

The NFκB pathway represents a distinct effector branch of the STING pathway [3, 4]. Other investigators have proposed that the type I interferon branch and the NFκB branch of the STING pathway are differentially regulated [5]. In these studies, TBK1 activation turned off IRF3 stimulation of type I interferon production. Our studies also demonstrated a distinction between the NFκB track and the type I interferon track of the pathway. For instance, STING expression levels correlated with downstream consequences of type I interferon production, but not with activation of the NFκB pathway.

In previous studies [14, 15, 16, 17, 18, 19, 20, 22], the bivariate relationships we found were mostly linear, meaning that an increase in the upstream molecule resulted in a commensurate increase in the downstream molecule. This rheostatic regulatory mechanism was seen in the type I interferon branch of the STING pathway with the linear relationships between STING and both BDNF and phospho‐Akt in the T cells and monocytes. These findings suggest appropriate manoeuvres for influencing the pathway. For these cells, transcriptional and/or translational regulation of STING expression may be crucial instead of a focus on STING phosphorylation. Conversely, in B lymphocytes, regulation of the type I interferon track was not correlated with STING expression but instead with phospho‐STING suggesting more of a focus on phosphorylation. Additionally, the relationship was logarithmic which indicates an on‐switch relationship instead of rheostatic.

The analysis of the relationships between the molecules at the bifurcation of the type I interferon and NFκB branches of the STING pathway also revealed nonlinear associations. All four mononuclear cell types demonstrated an inverse power relationship between phospho‐STING and phospho‐TBK1. The expression of one analyte was associated with the low expression of the other analyte. This configuration is an off‐switch mechanism. We were anticipating a positive interaction since these molecules have been shown to be mutually inducing [1, 6, 7, 8, 29, 34]. Using a short‐term stimulation with a STING agonist in culture, we did obtain the expected positive interaction, a logarithmic relationship with an on‐switch effect.

The inverse power association between phospho‐STING and phospho‐TBK1 in the clinical samples ex vivo may relate to the long‐term activation of the STING pathway in patients with coronary artery atherosclerosis. Prolonged stimulation of the STING pathway is known to activate a compensatory negative feedback mechanism to prevent persistent stimulation of the STING pathway [8]. The positive logarithmic relationship between these two molecules seen with in vitro stimulation may reflect short‐term effects without the induction of downregulation.

Evaluation of the phospho‐TBK1 and phospho‐RelA correlation revealed exponential relationships both in the study of blood mononuclear cells ex vivo and in vitro analysis with a STING agonist. This interaction represents an on‐switch that triggers progression to the NFκB pathway when a given threshold level of phospho‐TBK1 expression is reached.

Linear correlations (y = ax + b) among signalling pathway components are common. They represent rheostatic regulation of pathway flow. The presence of on‐off switches signified by nonlinear associations represents a unique mechanism to regulate signalling pathways. Examples of nonlinear associations seen in our study are logarithmic (y = a_*_ln(x) + b), exponential (y = ae^bx^) and power (y = ax^b^). Our finding of several nonlinear bivariate association among the molecules of the STING pathway suggests that these types of relationships may not be uncommon.

The advantage of mathematically defined nonlinear associations is the possibility of assessing them with an artificial intelligence algorithm. Current algorithms used in machine learning focus on the correlation or covariance matrix without reference to natural log transformation [35, 36, 37, 38, 39, 40, 41]. By including a correlation matrix of log‐normal transformed values versus non‐transformed values, investigators can assess both logarithmic and exponential relationships.

In our study, the inverse power association between phospho‐TBK1 and phospho‐STING was suggested by negative correlation coefficients in the non‐transformed correlation matrix. However, these coefficients were not uniformly impressive. The inverse power relationship was not appreciated until the bivariate plot was examined.

Our analysis of the STING pathway components does not provide directionality. For instance, we cannot determine by this analysis that activation of TBK1 induces phosphorylation of RelA. The prior experimental paradigm provided us with the directionality of the relationship. The integration of experimental studies with our observational analysis of clinical samples provides the most complete and most powerful picture of the STING pathway.

The evaluation of signalling pathways in blood samples from patients represents an important analytical capability that is likely to be powerful in understanding pathogenesis. The finding of nonlinear on‐off switches in signalling pathways, along with linear rheostatic mechanisms, provides a rich understanding of the regulatory mechanisms at play.

## Author Contributions

**David Kaplan:** conceptualization, methodology, investigation, validation, funding acquisition, project administration, writing – original draft, writing – review and editing, formal analysis, resources. Eric Christian: methodology, investigation, validation, writing‐ review and editing, formal analysis, resources.

## Funding

This study was funded by CellPrint Biotechnology LLC.

## Ethics Statement

All blood donations were obtained after informed consent. Sample collection and cellular analysis were approved by the Institutional Review Board of Case Western Reserve University School of Medicine. The study was conducted in accordance with the International Conference on Harmonisation Guidelines for Good Clinical Practice and the principles of the Declaration of Helsinki.

## Conflicts of Interest

The authors declare no conflicts of interest.

## Supporting information


**Figure S1.** Cell‐type‐specific bivariate relationships between phospho‐TBK1 and phospho‐GSK3β. Additional aliquots of samples from 55 patients were assessed for the expression of phospho‐TBK1, phospho‐RelA and phospho‐IKKε in CD4^+^ T cells and monocytes. The bivariate expressions of phospho‐TBK1 and phospho‐GSK3β are shown. Phospho‐IKKε and phospho‐GSK3β demonstrated significantly less correlation. The *p*‐value for the differences in r values (after the Fisher r‐to‐z transformation) for the correlation between phospho‐GSK3β and phospho‐TBK1 versus phospho‐GSK3β and phospho‐IKKε for CD4^+^ T cells is 0.012 and for monocytes is less than 0.0001.

**Table S1.** Phospho‐STING versus phospho‐TBK1 correlation coefficients.

## Data Availability

All data are available upon request to the corresponding author.
