# Supplementary figures and images for: Nonlinear Bivariate Associations and Mononuclear Cell‐Type‐Specific Expression Level Differences in the STING Signalling Pathway
Source: J Cell Mol Med. 2026 Mar 16;30(6):e71093. doi: 10.1111/jcmm.71093 (PMC13097638; doi:10.1111/jcmm.71093)

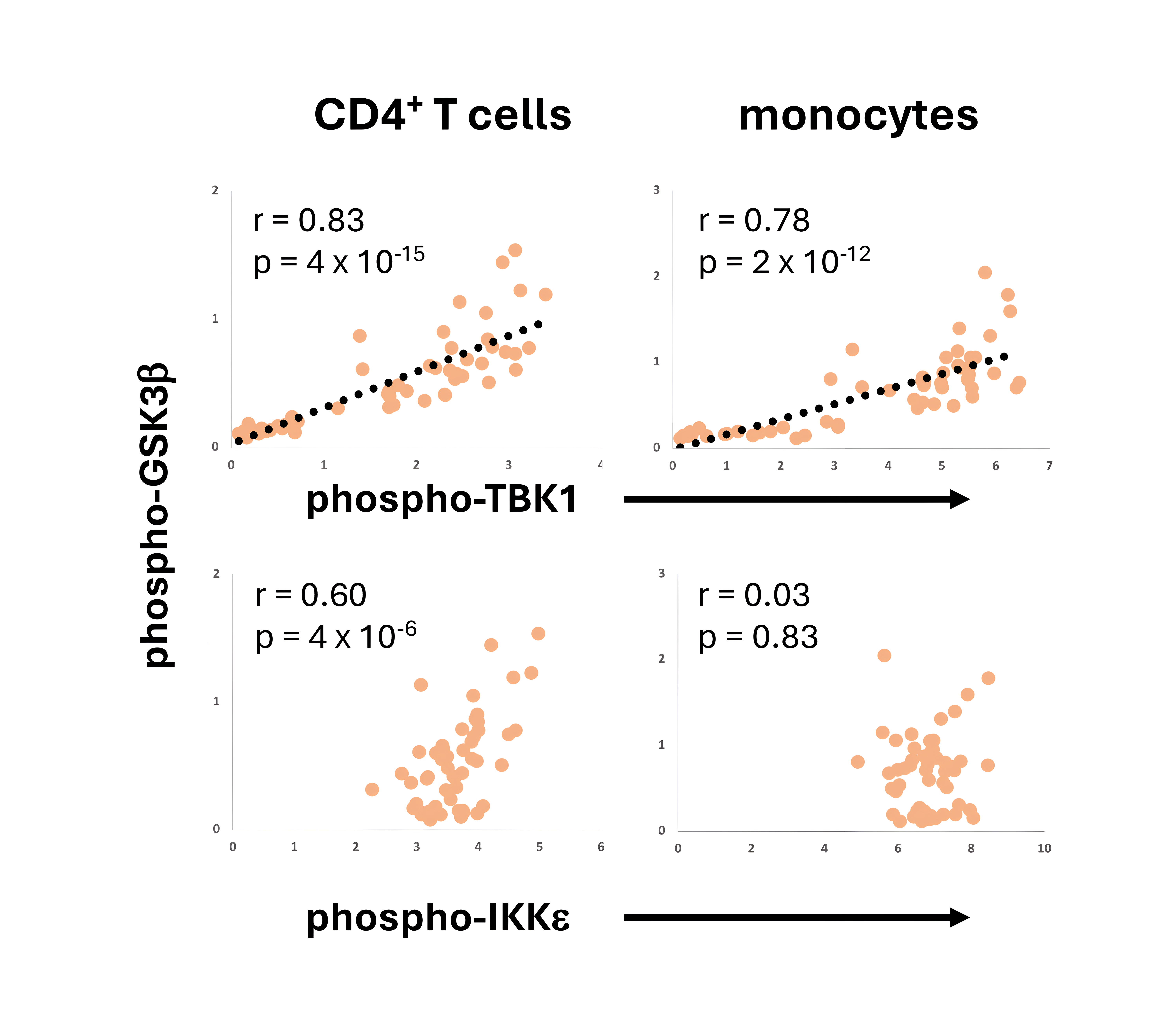

Supplement: Supplementary file 1 — Figure S1. Cell‐type‐specific bivariate relationships between phospho‐TBK1 and phospho‐GSK3β. Additional aliquots of samples from 55 patients were assessed for the expression of phospho‐TBK1, phospho‐RelA and phospho‐IKKε in CD4+ T cells and monocytes. The bivariate expressions of phospho‐TBK1 and phospho‐GSK3β are shown. Phospho‐IKKε and phospho‐GSK3β demonstrated significantly less correlation. The p‐value for the differences in r values (after the Fisher r‐to‐z transformation) for the correlation between phospho‐GSK3β and phospho‐TBK1 versus phospho‐GSK3β and phospho‐IKKε for CD4+ T cells is 0.012 and for monocytes is less than 0.0001. [file JCMM-30-e71093-s001.jpg]
