# Supplementary material for: Nonlinear Bivariate Associations and Mononuclear Cell‐Type‐Specific Expression Level Differences in the STING Signalling Pathway
Source: J Cell Mol Med. 2026 Mar 16;30(6):e71093. doi: 10.1111/jcmm.71093 (PMC13097638; doi:10.1111/jcmm.71093)
Supplement: Supplementary file 2 — Table S1. Phospho‐STING versus phospho‐TBK1 correlation coefficients. [file JCMM-30-e71093-s002.docx]

**Nonlinear bivariate associations and mononuclear cell-type-specific expression level differences in the STING signaling pathway**

David Kaplan*^^+^ and Eric L. Christian*

Supplemental Table 1

Phospho-STING versus phospho-TBK1 correlation coefficients

| **Cell-type** | **n** | **r value** | **p value** |
| --- | --- | --- | --- |
| CD4^+^ T cells | 115 | -0.50 | 10^-8^ |
| CD8^+^ T cells | 99 | -0.36 | 10^-4^ |
| B cells | 108 | -0.70 | 10^-15^ |
| monocytes | 115 | -0.42 | 10^-6^ |
